# Idiopathic Tetanus

**Published:** 1883-11

**Authors:** W. A. Cook

**Affiliations:** Atlanta, Ga.


ATLANTA
### Medical Register.

Vol. III.] NOVEMBER, 1883. [No. 2

### Original.

### IDIOPATHIC TETANUS.

By W. A. COOK, M. D., Atlanta, Ga.

As the existence of idiopathic tetanus is denied by a great many physicians, I thought a report of the following case might prove of some interest to your readers:

I was called in the afternoon of March 10, 1883, to see a colored hackman, aged 46. His wife informed me he had been treated by a physician for the past two days for cramp colic, but obtaining no relief she had dismissed him, and now wished me to take charge of his case.

She stated that he had been very much exposed to the imclement weather during the past three months, often being up all night.

On examination I found the following symptoms: All the muscles in the body were in a state of rigid contractraction; there was complete oposthotonos, the head and heels only touching the bed, the skin covering the forehead was corregated, the eyes fixed and prominent, sometimes suffused with tears, the nostrils were dilated, and the features fixed in a sort of grin—risus sardonicus.

The respirations were performed with difficulty and anguish. The pulse was frequent and feeble, and the skin was covered with perspiration; the jaws were firmly locked, and it was with difficulty that I could prie the mouth open sufficiently to give him medicine. He complained of great pain in his back and in the region of the heart. I diagnosed general idiopathic tetanus brought on from exposure to cold, as it was impossible to trace its origin to trumatism of any kind.

The patient having been freely purged with calomel before my arrival, I began the treatment by a hyperdemic injection of a half grain of morphine to relieve the intense pain from which he was suffering, and ordered 20 drops of chloroform to be given him every 2 hours.

March 11th.—Patient passed a restless night, but stated that the pain in back and chest was not so acute as the day before. There was no relaxation of the muscles except those of the neck, the opisthotonos not being so well marked as on the previous day.

There was still great difficulty in opening his mouth, but when once opened he could swallow his medicine with comparative ease. Another paroxysm of pain now having commenced, I gave him a half grain of morphine by hypodermic injection, and ordered the following prescription:

Quiniae sulph..................grs xxxvj.
Ext. belladon......................grs. xij.

M.—Ft. pills No. 12.

Sig.—Give one every 4 hours

I then ordered the chloroform to be increased to 30 drops every 4 hours.

March 12th.—Patient passed a more comfortable night, the cramps were not so prolonged nor so severe; he thought he had slept as much as 3 hours during the night.

The muscles of the neck and abdomen were somewhat relaxed, and the expression of countenance more natural.

I ordered him to continue the pills of quiniae and belladona, but to discontinue the chloroform.

I then gave him the following prescription:

℞.—Potass bromide chloralis hydratis......℥ j.
Tinct. hyosyam.......................℥ ij.
Water ad...........................℥ iv.

M.

Sig.—Take a teaspoonful every 2 hours.

March 13th.—Patient had slept well the past night. Pulse almost normal. All the muscles were relaxed except those of the jaws and legs

He could raise himself up in bed, but could not walk, owing to the contraction of the muscles of the legs. I ordered the prescription given him the day before to be continued, and instructed them to rub his jaws and legs well with chloroform liniment night and morning

March 14th.—Patient was entirely free from cramps, and had fully recovered the use of all the muscles except those of the jaws, which remained rigid for a week longer, after which time he recovered entire use of them

